# Intestinal Tuberculosis Presenting as an Ileocecal Mass in a Renal Transplant Patient

**DOI:** 10.7759/cureus.12995

**Published:** 2021-01-29

**Authors:** Jun Yang Jiang, Holly Greenwald, Vineet Gupta

**Affiliations:** 1 Medicine, University of California San Diego, San Diego, USA

**Keywords:** abdominal tuberculosis, renal transplantation, ileocecal mass

## Abstract

Abdominal tuberculosis accounts for approximately 5% of tuberculosis cases. However, recognition of this entity can be challenging in the absence of concomitant pulmonary involvement. Immunocompromised and immunosuppressed patients are at elevated risk for this infection and are confronted with increased side effects, drug interactions, and disease complications. We report the case of a 53-year-old female renal transplant recipient with a remote history of tuberculosis exposure who presented with sepsis and abdominal pain and was found to have an obstructive ileocecal mass. Serologic and pathologic testing ultimately led to the diagnosis of abdominal tuberculosis, and she was treated successfully with a course of antimycobacterial therapy with only minor complications.

## Introduction

Abdominal tuberculosis accounts for about 5% of tuberculosis cases and can affect the esophagus, stomach, intestinal tract, hepatobiliary system, pancreas, lymph nodes, and peritoneum [1]. The infection may occur as a result of direct ingestion, contiguous spread, hemolymphangitic spread, or reactivation of a latent infection [1,2]. Because symptoms vary based on host-pathogen interactions, diagnosis is not always straightforward, especially as pulmonary involvement is seen in fewer than 20% of patients [2]. Surgical or endoscopic exploration is often required to secure a tissue diagnosis [2].

Solid organ transplant recipients are at a significantly elevated risk for tuberculosis because of their immunosuppression (prevalence 0.3%-2.8%, 35 times that of the general population) and underlying comorbidities [3,4]. Tuberculosis must be distinguished from other infectious, inflammatory, and neoplastic etiologies. Mortality can reach 20%-30% despite treatment [3]. Here, we report the case of a renal transplant recipient who presented with fevers and abdominal pain and was diagnosed with intestinal tuberculosis after extensive laboratory, radiographic, and endoscopic evaluation.

## Case presentation

A 53-year-old Filipino woman with a history of lupus nephritis status post-renal transplantation presented with one month of right upper quadrant (RUQ) pain, lower abdominal pain, and fever. Her RUQ pain radiated to her back and worsened after meals, while her lower abdominal pain had no clear triggers. She reported no other constitutional symptoms, cough, hemoptysis, dyspnea, nausea, vomiting, bowel habit changes, or gastrointestinal bleeding. She had received a deceased donor kidney transplant seven years prior to her presentation and had had a stable allograft function. Her immunosuppressive regimen included prednisone, tacrolimus, and mycophenolate mofetil. She immigrated from the Philippines to the United States at age 16 and visited Los Cabos, Mexico, four months prior to presentation. She did not report any sick contacts.

**Figure 1:**
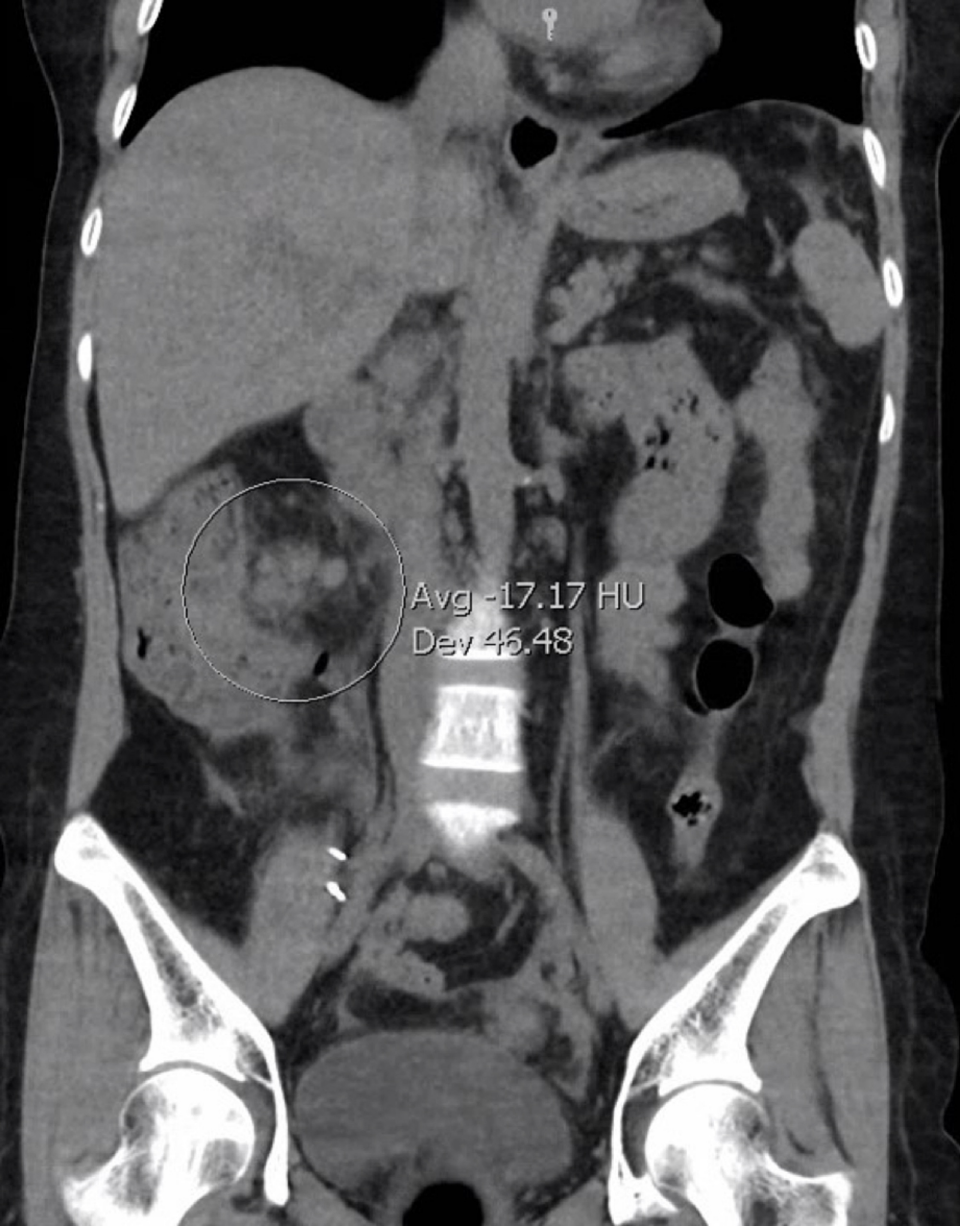
CT abdomen/pelvis showed marked thickening of the terminal ileum and prominent ileocolic lymphadenopathy.

At presentation, she was febrile to 38.5°C with a heart rate of 104 and respiratory rate in the mid-20s. Physical examination was notable for RUQ and hypogastric tenderness. Laboratory studies were remarkable for a leukocyte count of 13.2 × 109/L (reference range [RR]: 4-10 × 109/L), hemoglobin of 10.9 g/dL (RR: 11.2-15.7 g/dL), bicarbonate of 18 mmol/L (RR: 22-29 mmol/L), blood urea nitrogen of 26 mg/dL (RR: 6-20 mg/dL), creatinine of 1.49 mg/dL (RR: 0.51-0.95 mg/dL), and alkaline phosphatase 425 units/L (RR: 35-140 units/L). CT abdomen/pelvis without contrast on admission showed terminal ileitis with prominent lymphadenopathy, cholelithiasis with gallbladder wall edema, and bilateral pleural effusion (Figure 1).

She was empirically started on vancomycin and piperacillin/tazobactam. As her blood and stool studies were finalized as negative on hospital day 3, the antibiotics were stopped. However, she developed a recurrent fever to 39.3°C, and repeat CT on hospital day 5 demonstrated worsening terminal ileitis and bulky regional lymphadenopathy concerning for colon adenocarcinoma or post-transplant lymphoproliferative disease (PTLD).

**Figure 2:**
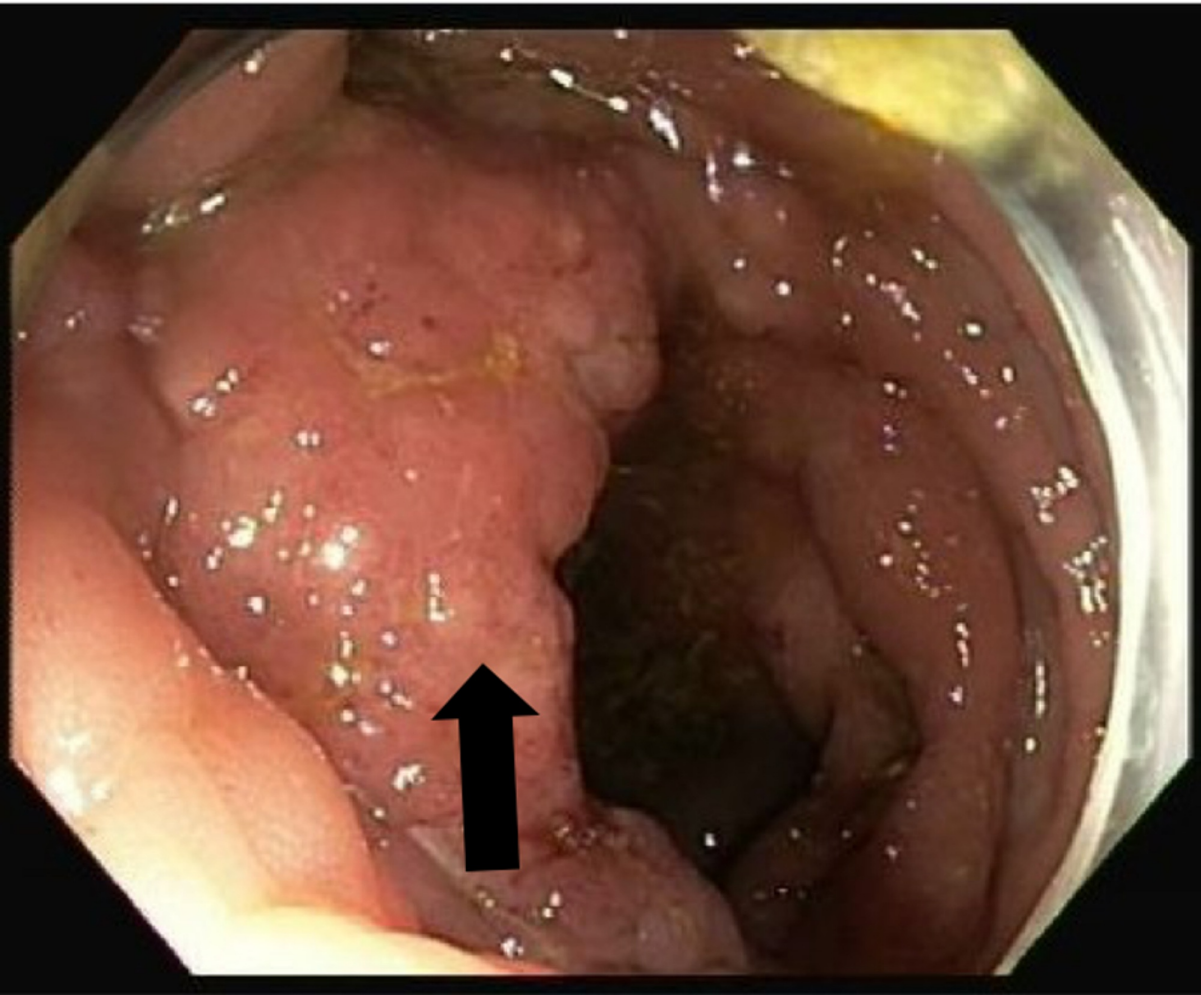
Colonoscopy reveals an ileocecal mass

Subsequent colonoscopic examination disclosed a large, protruding, nodular mass and ulcer at the ileocecal valve (Figure 2).

**Figure 3:**
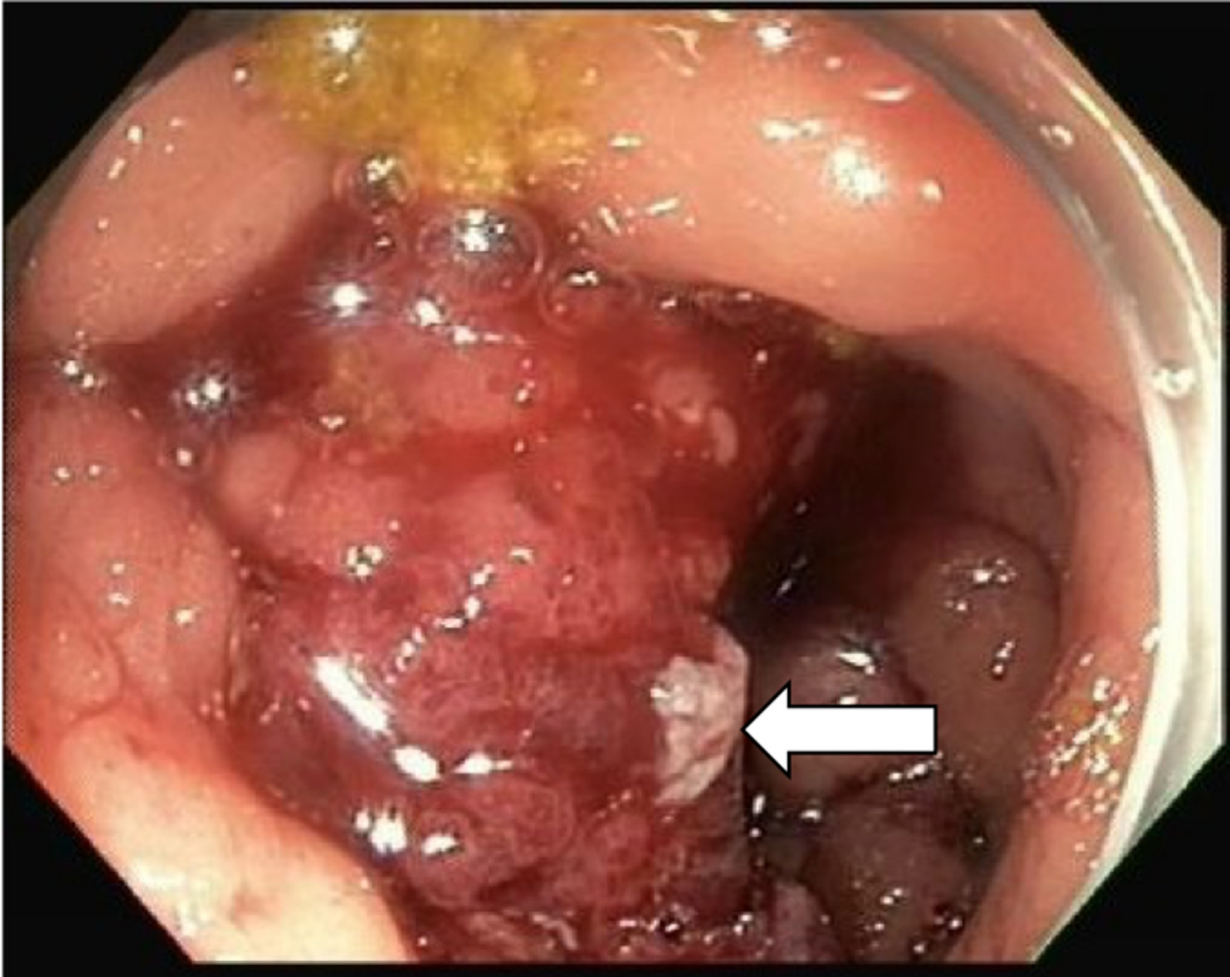
Colonoscopy reveals an ascending colon ulcer

As the mass obstructed the inlet to the terminal ileum, the colonoscope could not be advanced past the ileocecal valve. A small ulcer was also seen in the ascending colon (Figure 3).

**Figure 4:**
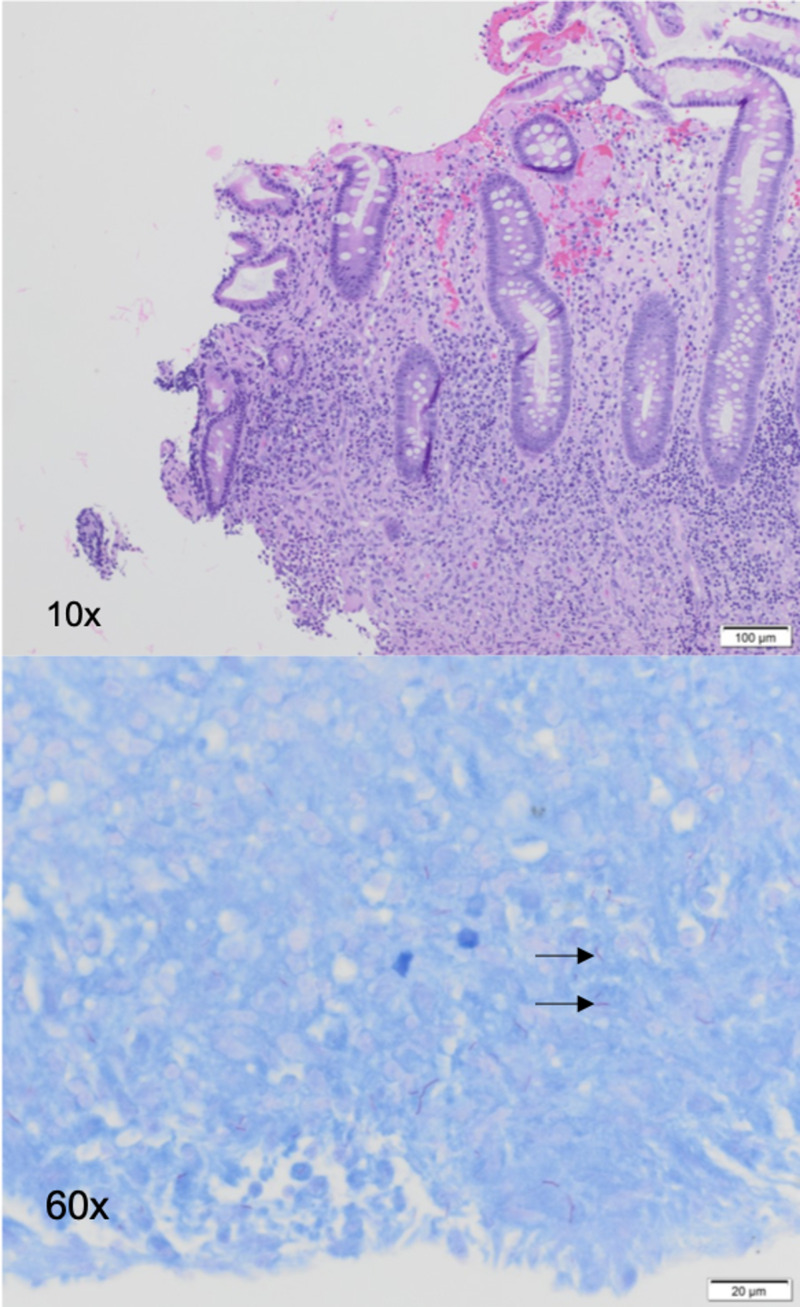
Ileocecal valve biopsy shows extensive granulomatous inflammation with AFB (top: 10×, hematoxylin and eosin stain; bottom: 60×, acid-fast stain) consistent with mycobacterial infection. AFB: Acid-fast bacillus

The mass and ulcer were biopsied, and specimens were sent for histological examination. A stool acid-fast bacillus (AFB) smear and an interferon-gamma release assay for Mycobacterium tuberculosis response were positive. Pathology from the colonoscopy showed active ileocolitis, extensive caseating granulomas, and numerous AFB (Figure 4).

Given the constellation of laboratory, radiographic, and endoscopic findings, the patient was diagnosed with intestinal tuberculosis [5]. A stool culture and one out of three induced sputum cultures eventually grew Mycobacterium tuberculosis.

Rifabutin, isoniazid, pyrazinamide, and ethambutol (RIPE) were promptly started after the results of pathology examination returned. The patient was discharged on hospital day 20 with close outpatient follow-up and direct observed therapy at home as her abdominal symptoms and laboratory studies began to improve. After two months of RIPE therapy, the regimen was reduced to rifabutin and isoniazid alone. However, her serum creatinine climbed steadily over the next six months. A biopsy of the transplanted kidney showed acute worsening of her chronic cell-mediated rejection. An oral prednisone taper was begun with subsequent improvement in allograft function.

## Discussion

Tuberculosis should be a differential diagnosis in all patients with subacute gastrointestinal complaints and a plausible prior exposure. This may be challenging in settings with relatively low burden of tuberculosis [5]. While tuberculosis had been considered since the outset for our patient, her acute presentation, concomitant biliary disease, and minimal pulmonary involvement compelled us to consider alternative etiologies. However, the persistence of fevers and ileocecal mass on otherwise appropriate antibacterial agents ultimately led to an endoscopic biopsy and a tissue diagnosis.

When intestinal tuberculosis is found, the ileocecal region is implicated in 75%-84% of cases [2,6]. Patients often report fever, anorexia, and changes in bowel habits [2]. In immunocompromised individuals, however, ulcerative symptoms are more common, and obstruction can be seen on rare occasions [3]. Endoscopic biopsy is indicated to distinguish intestinal tuberculosis from other causes of terminal ileitis, which include Crohn’s disease, colonic adenocarcinoma, and lymphoma (e.g., non-Hodgkin lymphoma, PTLD) [3,6]. Studies suggest that the traditional, six-month RIPE regimen appears to have similar cure and relapse rates for abdominal tuberculosis as an extended, nine-month course [7].

Antimycobacterial therapy in transplant recipients poses a unique set of challenges: increased side effects, drug interactions, and disease complications. In our patient, rifampicin, part of the traditional RIPE regimen, depressed serum tacrolimus levels and was replaced with rifabutin, which bears a lower risk of inducing the cytochrome P450 system [8,9]. Her fluctuating tacrolimus levels and withheld mycophenolate mofetil nevertheless caused acute cell-mediated rejection of her transplant kidney; a steroid taper was given to curb the further deterioration of her allograft function. Despite these obstacles, the patient completed therapy, and her gastrointestinal symptoms resolved.

## Conclusions

Intestinal tuberculosis should be considered in a differential diagnosis for all patients with subacute gastrointestinal complaints and possible prior exposure. This may be challenging in patients with atypical presentation of tuberculosis, conflicting comorbidities especially in settings with a low burden of tuberculosis, warranting a high index of suspicion for a timely intervention.
